# Risk Assessment of Mercury in Soil, Water, and Sediments Collected in a Region Impacted by Gold Mining

**DOI:** 10.1021/acsomega.4c09617

**Published:** 2025-02-20

**Authors:** Saulo V. A. Dantas, Caio S. A. Felix, Francisco A. S. Cunha, Jailson B. de Andrade, Sergio L. C. Ferreira

**Affiliations:** † Universidade Federal da Bahia, Instituto de Química, Campus Ondina, Salvador 40170-270, Bahia, Brazil; ‡ Universidade Federal da Bahia, Instituto Nacional de Ciência e Tecnologia de Energia & Ambiente, INCT, Salvador 40170-115, Bahia, Brazil; § Centro Interdisciplinar de Energia & Ambiente, Universidade Federal da Bahia, CIEnAm, Salvador 40170-115, Bahia, Brazil; ∥ Centro Universitário SENAI, CIMATEC, Avenida Orlando Gomes, 1845, Salvador 41650-000, Bahia, Brazil

## Abstract

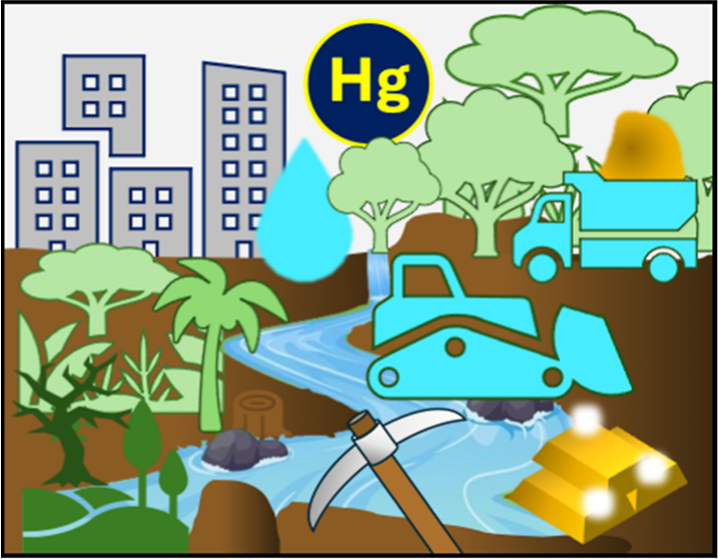

This work reports the determination of mercury and the assessment of health risks in water, river sediments, and soil samples collected in the city of *Jacobina*, Brazil, located in a region impacted by gold mining. The mercury determinations were performed using the direct method analyzer (DMA) method, which was carefully validated according to the IUPAC recommendations. Total mercury contents ranged from 0.10 to 1.77 μg L^–1^ in water samples, from 0.005 to 0.278 μg g^–1^ in soil samples, and from 0.014 to 0.317 μg g^–1^ in river sediment samples. The contamination factor (CF) and the ecological risk index were applied to the data obtained from soil and sediment analyses. Only one of the twenty-one soil samples had CF > 1 (1.11), denoting moderate contamination for this sampled site. All 20 other samples had CF < 1 (0.02 to 0.69), showing low mercury contamination in the soils collected throughout *Jacobina*. The results of the ecological risk index perfectly corroborated the results found by the CF, denoting that only one sample had a moderate potential ecological risk. Among the 13 sediment samples, two had CF > 1 and Er in the range of 40 to 80, denoting moderate contamination and moderate ecological risk in these two sampled locations. All 11 other samples had CF < 1 and Er less than 40, demonstrating that the sediments analyzed have low contamination and low potential for ecological risk due to mercury. All mercury levels found in water samples are below the maximum limit (2 μg L^–1^) established by the CONAMA, the Brazilian Government, for class 3 freshwater. The assessment of daily mercury exposure was also performed using the routes of exposure through dermal contact (ADIder), inhalation (ADIinh), ingestion (ADIing) in the soil samples, and dermal contact and ingestion in the water samples employing the health risk indices established by the USEPA. The results obtained did not indicate noncarcinogenic risks for adults and children.

## 1 Introduction

The city of *Jacobina* in Bahia has been mining gold ore since 1720 and is one of the leading Brazilian municipalities that have extracted this noble metal.^1^ The town has the Itapicuru-Mirim and Ouro rivers and 48 waterfalls distributed throughout the municipality, with an average population of 84 thousand. The Itapicuru-Mirim River, with a length of more than 40 km, was widely used in the past for domestic and other activities by the population. The gold mining complex (consisting of five mines and two tailings dams) also uses this water resource in the industrial process. The Itapicuru-Mirim river establishes the Itapicuruzinho dam, and its water is used to supply the city of *Jacobina*. Although mining activities are old, only one work was performed to evaluate the impact of using mercury in the gold process. In 2013, five samples of surface sediments were collected from the Itapicuru-Mirim river located in *Jacobina* city, and the chemical elements arsenic, cadmium, chromium, copper, iron, mercury, manganese, nickel, lead, and zinc were quantified using inductively coupled plasma optical emission spectrometry. The authors concluded that the mercury levels found in the sediment samples are due to anthropogenic sources, ranging from <0.01 to 0.36 μg g^–1^ in fraction <1.5 mm and from 0.19 to 0.44 μg g^–1^ in fraction <75 μm.^2^

Traditionally, gold mineralization processes require mercury, whose toxic nature usually causes contamination problems in soil, water bodies, and the atmosphere in regions close to the mining companies.^3−7^ Mercury concentrations were quantified in sediments from the Atrato River basin, which was heavily impacted by gold mining.^3^ Mercury levels ranged from 0.09 to 0.23 mg kg^–1^, with the highest values found in regions affected by gold mining. In this work, the contamination factor (CF), pollutant load index, geoaccumulation index (Igeo), and ecological risk factor were used to characterize the contamination and pollution in the region under study. Total mercury and methylmercury were quantified in water, sediments, and fish from lagoons near an abandoned gold mining site in western Colombia. In fish samples, mercury was found mainly as methylmercury (83–95%); total mercury concentrations in water samples showed values above the EPA limit, highlighting a critical contamination situation.^4^ A study evaluated mercury contamination resulting from artisanal gold mining in Ghana. Two hundred and thirty-seven soil samples were collected from different locations. Total mercury concentrations in soils reached an average value of 71 mg kg^–1^ in active mining sites and 2.7 mg kg^–1^ in moderate mining regions. Enrichment factor (EF) was used to classify the contamination in the collected soil samples.^5^ Researchers evaluated the distribution and bioavailability of mercury in sediments collected on Ceram Island, Indonesia, an area influenced by gold mining. The levels found revealed a major environmental problem in the studied area.^6^

The contamination from chemical elements in soils and sediments has been assessed using pollution and ecological risk indices.^8−10^ The Igeo,^11^ EF,^12^ and pollution index (PI)^13^ have been widely used.^8^ However, these indices cannot be calculated in some cases due to the lack of background values for the chemical elements in the studied region. On the other hand, the CF and the ecological risk index (Er) are contamination assessment parameters calculated based on the concentration of the chemical elements in the preindustrial period, following the consistent work published by Hakanson in 1980.^14^

The levels of chemical elements in soil samples also make it possible to assess the carcinogenic and noncarcinogenic risk of adults and children, considering the exposure routes^15,16^ of dermal absorption, inhalation, and ingestion.^17−22^ A researcher collected analytical data from soils from thirty-two Indian cities of works published between 2001 and 2019 to estimate the contamination of the chemical elements arsenic, chromium, copper, zinc, nickel, and lead. The data obtained were evaluated using carcinogenic and noncarcinogenic risks, demonstrating potential cancer risks for chromium and arsenic for adults and children.^17^ Chen et al. collected 90 soil samples in the mercury mining/smelting region of Tongren, southwest China. The ecological risk index for mercury denotes potential risks for most samples collected in the mining region. However, the noncarcinogenic risks for these same soils were acceptable. Risks relating to ingestion, dermal, and inhalation routes were also reported.^18^ Researchers from Ethiopia collected soil samples from a vegetable-growing region and determined the concentrations of nine chemical elements to assess the contamination. Target hazard quotient (THQ) was applied to the results obtained, denoting adverse health risks arising from arsenic, lead, mercury, and cobalt in children and lead and cobalt in adults.^19^ Mercury was quantified in 12 soil and 7 water samples from a gold mining region in Abu Hamad, Sudan. Human health risk indices were applied to the results obtained, with the inhalation exposure route presenting the highest THQ for all soil samples analyzed. However, the water samples presented a low risk of contamination.^21^ Surface sediment samples from a mangrove were collected in the commercial area of Zhanjiang Bay, China, and the concentrations of cobalt, vanadium, copper, lead, nickel, arsenic, cadmium, and mercury were quantified using ICP–MS. Risk assessment indices were applied to the results, denoting that all elements presented low contamination factors, except for cadmium, copper, and mercury. Furthermore, cadmium presented a high potential ecological risk, followed by mercury and copper.^22^

This work reports an assessment of the impact of mercury used in gold mining in the city of *Jacobina*. Soil, water, and river sediment samples were analyzed by using the direct method analyzer (DMA) method. The CF and ecological risk indices were applied to the analytical data to characterize the contamination in the region studied.

## 2 Materials and Methods

### 2.1 Sample Collection

Samples were collected near the mining area, around the city’s dam, in the municipal park, and along rivers in regions with varying levels of human activity. Sediment and soil matrices were taken from 5 cm of the surface layer, and water samples were taken from 30 cm deep. Soil and sediment samples were collected using a steel shovel and a spatula, respectively, which were rigorously washed and decontaminated after each sampling step. Soils were collected after vegetables, stones, and other residues were removed from the sampled location.

The following samples were collected: 21 soil samples, 13 sediment samples, 6 samples collected from the Itapicuru-Mirim river (SD1 to SD6), 4 in the do Ouro river (SD7 to SD10), 3 samples collected after the confluence of the do Ouro river in the Itapicuru-Mirim river (SD11 to SD13), and 19 water samples collected in different regions of *Jacobina* city. All of these samples were collected in December 2023.

**Figure 1:**
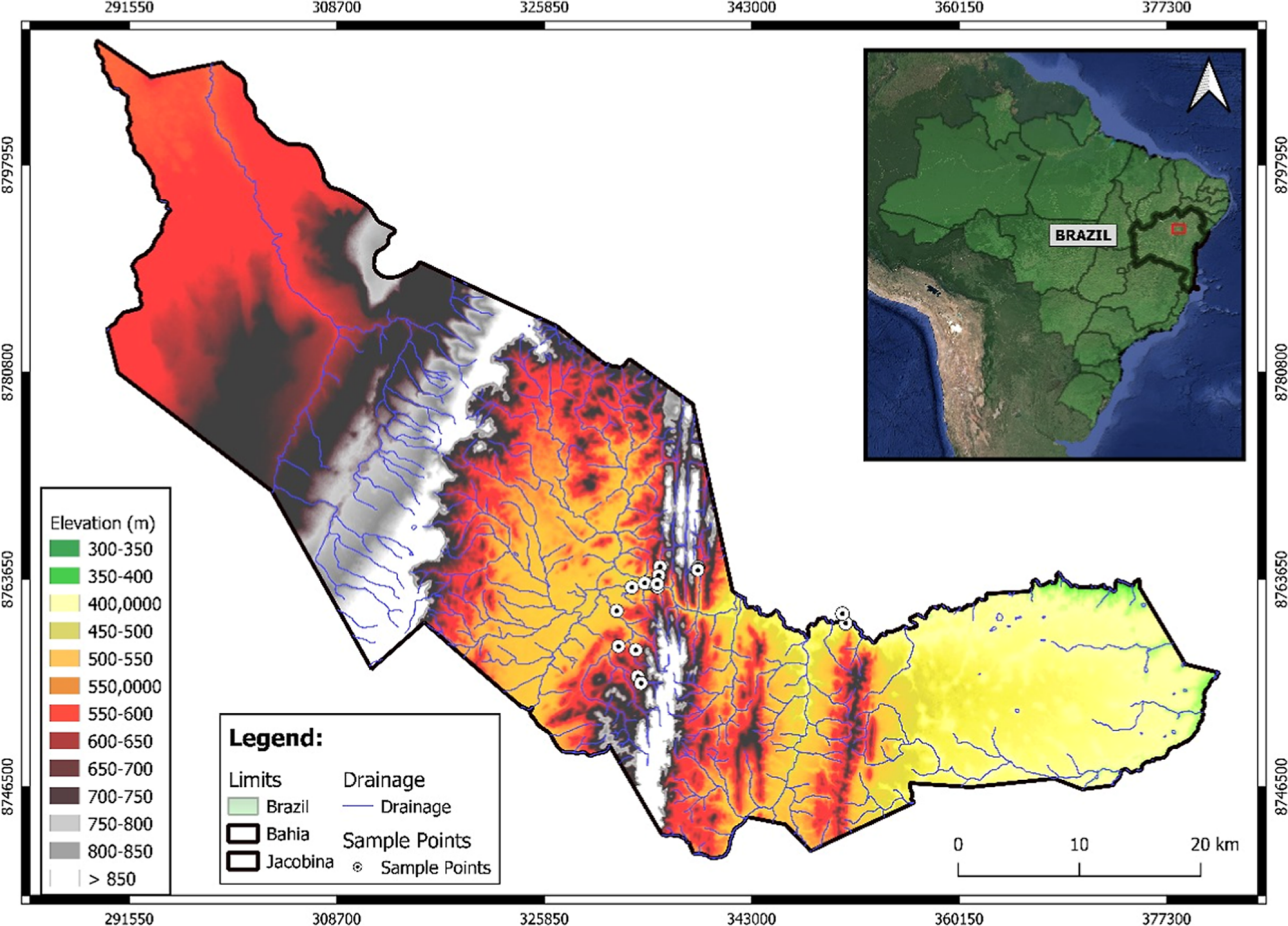
Altimetric distribution of the *Jacobina* city in Bahia, Brazil.

The map shown in Figure 1 reveals the altimetric distribution of *Jacobina* city in Bahia, highlighting specific sample collection points about the local relief. The region has significant elevation variation, with altitudes ranging from 300 m in the lowest areas to more than 850 m in the upper regions, as indicated on the map in white. The collection points, indicated by white circles, are mainly located in the central and northeast areas of the city, which have an intermediate elevation (between 500 and 700 m). These collection points are located in areas close to drainages (rivers and streams), indicating the interest in analyzing the influence of elevation and proximity to watercourses on the soil quality, water, and sediment in the region. Additionally, the map includes information about the city’s local hydrography and geographic limits, indicating the notable presence of rivers throughout the territory.

### 2.2 Sample Processing

Around 150 g of soil and sediment samples were collected and stored in glass beakers. Afterward, they were processed on an open mesh sieve to exclude residues from the collection. Also, they were freeze-dried for 72 h at a temperature of −50 °C and a vacuum pressure of 0.99 bar, with the aim of ensuring complete moisture removal. After being freeze-dried, samples were lightly ground using a porcelain mortar and pestle. Finally, in the grinding step, the material was processed on an 80 mesh sieve (pore size 177 μm) to facilitate obtaining a homogeneous matrix.

Sample volumes of 200 mL of water were collected in glass bottles, followed by a pH adjustment to less than 2.0 by using concentrated nitric acid. The transport to the laboratory was performed using a closed thermal box, and the bottles were kept on ice. In the laboratory, the samples were filtered using 0.45 μm cellulose acetate membrane filters and finally stored at 4.0 °C ± 2 °C until analysis using the DMA method. The bottles used for the sampling were previously washed and decontaminated using a 10% nitric acid solution and washed with ultrapure water. The nitric acid used to preserve the samples was double-distilled to avoid contamination and reduce the method’s quantification limit.

### 2.3 Determination of Mercury in Water, Sediment, and Soil Samples—Instruments

A water purification system from a Millipore Direct-Q8 UV (Millipore, Bedford, MA, USA) was employed to obtain ultrapure water (ASTM type I) with a resistivity of 18.2 MΩ cm. The ultrapure nitric acid used for pH adjustment was obtained from a Milestone duoPUR quartz sub-boiling distillation system (Milestone, Sorisole, Italy). Soil and sediment samples were freeze-dried by using the SL-404/B freeze-dryer (Solab, Piracicaba, Brazil). Mercury determinations in water, sediment, and soil samples were performed by employing the DMA-80 Tri Cell Direct Mercury Analyzer [Milestone, Sorisole (BG), Italy].

### 2.4 Quality Control

Total mercury was determined in three matrices (soil, water, and river sediment) using calibration curves in the range of 0.01–100 ng of inorganic mercury, which allowed limits of detection (LD) and quantification (LQ) of 4.0 and 12.0 pg, respectively. These limits were calculated as LD = (3o′/S) and LQ = (10o′/S), respectively, (o′) being the standard deviation of measurements of 10 blank solutions and (S) the slope of the calibration curve. Intraday and interday tests were performed to evaluate the precision, calculating the coefficients of variation obtained by determining ten replicates of a soil sample. The results obtained for interday and intraday were 2.7% and 11.39%, respectively. The accuracy of the DMA method was confirmed by analyzing the certified reference material of marine sediment furnished by the National Research Council of Canada (PACS-2). The results obtained by the DMA method was (2.91 ± 0.33 μg g^–1^), which agrees with the certified value of (3.04 ± 0.20 μg g^–1^). During the experimental development of the work, all calibration curves had coefficients of determination of greater than 0.999. Sample masses used for mercury determination were 100 mg for soil and 25 mg for sediment.

### 2.5 Contamination Factor and Ecological Risk Indices

The CF is calculated by the relationship between the concentration of the chemical element present in the soil expressed in μg g^–1^ and the concentration of this element in the preindustrial period, which for mercury is 0.25 μg g^–1^.^8,9,14^ The CF values allow for classifying the contamination level of soils and sediments as low (CF < 1), moderate (1 ≤ CF < 3), considerable (3 ≤ CF < 6), and very high (CF ≥ 6).^14^

The ecological risk index (Er) of a chemical element of the soil is determined by multiplying the CF by the toxic-response factor of the element. For mercury, the toxic-response factor is 40. The Er values classify the soils as follows: low risk (Er < 40), moderate risk (40 ≤ Er < 80), considerable risk (80 ≤ Er < 160), high risk (160 ≤ Er < 320), and severe risk (Er ≥ 320).^8,9,14^

### 2.6 Application of Human Health Risk Indices in Soil Samples

The noncarcinogenic risk (HI) from metal in the soil is assessed using the target risk quotient (HQ) index, which estimates three routes of exposure, which are dermal absorption (ADIder), inhalation (ADIinh), and oral ingestion (ADIing).^15,23,24^ The following equations calculate the risks
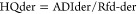
1
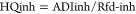
2
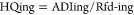
3
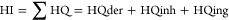
4where ADIder, ADIing, and ADIing are the average daily exposure doses (mg/kg/day) of the chemical element through dermal absorption, inhalation, and oral ingestion, respectively, whose calculations are established by the equations
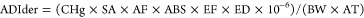
5
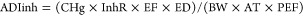
6
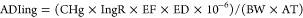
7

RfDs are the reference doses of the chemical elements. The values for mercury are 0.000021, 0.0000857, and 0.0003 (mg/kg/day) for RfD-der, RfD-inh, and RfD-ing, respectively.^15,23,24^

HI values classify the noncarcinogenic risk as follows: HI ≤ 1 denotes that there is no risk; HI > 1 denotes that there is a likelihood of noncarcinogenic risk. The parameters used to calculate the noncarcinogenic risk (HI) index are shown in Table 1.

**Table 1:** Parameters Used to Determine the Noncarcinogenic Risks

parameters	definition	units	adult	children
C_Hg_	mercury concentration	μg g^–1^		
Cw(Hg)	mercury concentration	μg L^–1^		
BW	body weight	kg	70	15
ED	exposure duration	year	30	6
EF	exposure frequency	day year^–1^	350	350
AT	averaging time	day	ED × 365	ED × 365
SA	exposed skin surface area	cm^2^	5700	2800
AF	skin adherence factor	mg cm^–2^	0.07	0.2
ABS	dermal absorption factor		0.001	0.001
PEF	particular emission factor	m^3^ kg^–1^	1.36 × 10^9^	1.36 × 10^9^
InhR	inhalation rate	m^3^ day^–1^	20	7.6
IngR	ingestion rate	mg day^–1^	100	200
ET	water exposure time	h event^–1^	0.58	0.58
IngRwater	water Ingestion rate	L day^–1^	2	2
*K*p	permeability coefficient	cm h^–1^	1 × 10^–3^	1 × 10 ^–3^
CF	conversion factor	L cm^–3^	1/1000	1/1000
ABSw	dermal absorption factor (water)		0.1	0.1

### 2.7 Application of Human Health Risk Indices in Water Samples

In addition, the noncarcinogenic risk (HI) from mercury in water is evaluated by the target risk quotient (HQ) index,^15,24−26^ estimating the routes of exposure by dermal absorption (ADIderw) and oral ingestion (ADIingw),^27^ which are calculated with the following equations
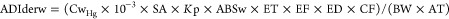
8
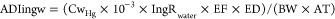
9
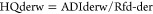
10
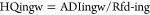
11
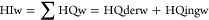
12

The reference doses’ values are the same as those used for evaluation in soil samples. Table 1 also shows the parameters used to calculate these indices. Dermal absorption refers to the process of absorbing substances through the skin, while oral ingestion refers to taking substances into the body through the mouth.

## 3 Results and Discussion

### 3.1 Assessment of Contamination of Soil Samples Collected in *Jacobina*, Brazil

The DMA method with detection and quantification limits of 4 × 10^–5^ and 1.2 × 10^–4^ μg g^–1^, respectively, was applied to determine total mercury in twenty-one surface soils collected in different regions of *Jacobina*. Table 2 shows that mercury concentrations ranged from 0.005 to 0.278 μg g^–1^, with mean and median values of 0.088 and 0.091 μg g^–1^, respectively. The CF is determined by the relationship between the mercury concentration in the sample investigated and the mercury concentration (0.25 μg g^–1^) in the preindustrial period, according to Hankanson.^14^ The product of the CF and the toxic response factor calculates the ecological risk, given that the response factor for mercury is 40.^15^

**Table 2:** Determination and Risk Assessment of Mercury in Surface Soils from *Jacobina* City

sample	Hg concentration (μg g^–1^)	CF	Er
SL1	0.090 ± 0.001	0.36	14.5
SL2	0.151 ± 0.006	0.60	24.1
SL3	0.157 ± 0.005	0.63	25.2
SL4	0.174 ± 0.021	0.69	27.8
SL5	0.091 ± 0.005	0.36	14.5
SL6	0.091 ± 0.008	0.37	14.6
SL7	0.035 ± 0.005	0.14	5.6
SL8	0.128 ± 0.007	0.51	20.5
SL9	0.164 ± 0.018	0.65	26.2
SL10	0.040 ± 0.004	0.16	6.4
SL11	0.058 ± 0.002	0.23	9.3
SL12	0.036 ± 0.003	0.15	5.8
SL13	0.100 ± 0.007	0.40	16.0
SL14	0.004 ± 0.001	0.02	0.6
SL15	0.005 ± 0.001	0.02	0.8
SL16	0.278 ± 0.029	1.11	44.4
SL17	0.042 ± 0.002	0.17	6.8
SL18	0.015 ± 0.002	0.06	2.3
SL19	0.018 ± 0.001	0.07	2.9
SL20	0.055 ± 0.009	0.22	8.8
SL21	0.120 ± 0.007	0.48	19.1

The pollution level of the soil samples was evaluated by using the CF and ecological risk indices. The results demonstrated that only one sample located in a region impacted by clandestine mining with an average value of mercury of 0.278 μg g^–1^ had a CF greater than 1 (1.11) and ecological risk in the range of 40 to 80 (Er 44.4), denoting moderate contamination and moderate ecological risk for this region. The SL2 sample collected in the waste disposal region in the river presented a concentration higher than the average found, 0.151 μg g^–1^, but the CF and Er values showed low contamination and ecological risk. The SL20 sample, collected at the main mining gate, showed low contamination and ecological risk, with an average mercury concentration of 0.055 μg g^–1^. A general assessment of the data obtained shows low mercury contamination in the soils from *Jacobina*, Brazil. All the CF and Er values are shown in Table 2.

### 3.2 Assessment of Contamination of Sediment Samples Collected in *Jacobina*, Brazil

The mercury determination in the surface sediment samples by the DMA method was done with LD and Loq of 1.6 × 10^–4^ and 4.8 × 10^–4^, respectively. Thirteen samples were analyzed: six collected in the Itapicuru-Mirim river (SD1 to SD6), four in the do Ouro river (SD7 to SD10), and three after the confluence of the do Ouro river in the Itapicuru-Mirim river (SD11 to SD13). As shown in Table 3, total mercury concentrations ranged from 0.014 to 0.317 μg g^–1^, with median and mean values of 0.080 and 0.107 μg g^–1^, respectively. The highest concentrations of mercury were found at collection points SD3 (0.317 μg g^–1^) and SD1 (0.272 μg g^–1^), whose contamination factors and ecological risks were 1.27 and 50.7 for SD3 and 1.09 and 43.5 for SD1, denoting moderate contamination and moderate potential ecological risk. Point SD1 is located at the Itapicuru-Mirim river dam, and SD3 is a commercial region with a large flow of people. Point SD2 has an average total mercury value of (0.111 μg g^–1^), with (CF 0.44) and (Er 17.8) located in the mining company’s waste disposal region, highlighting an area of low contamination and ecological risk. All other CF and Er values found in sediment samples from the de Ouro river and Itapicuru-Mirim river denoted low contamination and low ecological risk. All of the contamination factors and ecological risk values are shown in Table 3.

**Table 3:** Determination and Risk Assessment of Mercury in Sediments from *Jacobina* City

samples	Hg concentration (μg g^–1^)	CF	Er
SD1	0.272 ± 0.018	1.09	43.5
SD2	0.111 ± 0.009	0.44	17.8
SD3	0.317 ± 0.046	1.27	50.7
SD4	0.198 ± 0.023	0.79	31.7
SD5	0.113 ± 0.022	0.45	18.1
SD6	0.016 ± 0.001	0.06	2.6
SD7	0.014 ± 0.002	0.06	2.2
SD8	0.014 ± 0.001	0.06	2.6
SD9	0.171 ± 0.037	0.68	27.4
SD10	0.029 ± 0.007	0.12	4.6
SD11	0.080 ± 0.019	0.32	12.8
SD12	0.019 ± 0.001	0.08	3.0
SD13	0.042 ± 0.001	0.17	6.7

### 3.3 Assessment of Contamination of Water Samples Collected in *Jacobina*, Brazil

Determining total mercury in the 19 surface water samples collected in *Jacobina* was also performed using the DMA method with detection and quantification limits of 0.008 and 0.024 μg L^–1^, respectively. Table 4 shows that the total mercury contents ranged from 0.10 to 1.77 μg L^–1^, with median and mean values of 0.26 and 0.41 μg L^–1^. The maximum value (1.77 μg L^–1^) was found in a well with a depth of 10 m located in an agricultural region. All total mercury contents found are below the maximum limit established by the Brazilian Government, which is 2.0 μg L^–1^ for class 3 fresh waters, whose destinations include supply for human consumption after conventional or advanced treatment; irrigation of trees, cereal, and forage crops; amateur fishing; secondary contact recreation; and watering of animals.

**Table 4:** Mercury Determination in Surface Water Samples from *Jacobina* City

samples	Hg concentration (μg g^–1^)
A1	0.55 ± 0.14
A2	0.60 ± 0.06
A3	0.85 ± 0.16
A4	1.77 ± 0.05
A5	0.56 ± 0.05
A6	0.41 ± 0.12
A7	0.36 ± 0.03
A8	0.22 ± 0.06
A9	0.31 ± 0.10
A10	0.39 ± 0.20
A11	0.18 ± 0.03
A12	0.15 ± 0.04
A13	0.19 ± 0.07
A14	0.32 ± 0.22
A15	0.20 ± 0.14
A16	0.26 ± 0.11
A17	0.26 ± 0.11
A18	0.14 ± 0.01
A19	0.10 ± 0.03

### 3.4 Noncarcinogenic Risk Assessment in Soil Samples

The noncarcinogenic risks (HI) arising from the total mercury concentrations in the 21 soil samples analyzed were calculated using the target risk quotient (HQ) index following the three routes of exposure, which are dermal absorption (ADIder), inhalation (ADIinh), and oral ingestion (ADIing).^15,23,28^ The equations used to calculate these indices are detailed in item 2.4, and the parameters established by the USEPA for the calculations are shown in Table 1. The daily Hg exposure was evaluated for both age groups: adults and children. The results in Table 5 demonstrated that all the HI values are lower than 1.0, denoting no noncarcinogenic health risks for soil samples analyzed. In addition, the classification of the exposure routes follows the order HQing > HQderm > HQinh for both children and adults. In general, the values found for children show greater vulnerability for this type of exposure assessment.^15,17,23^

**Table 5:** Noncarcinogenic Risk Assessment in the Soil Samples from *Jacobina*, Brazil

soil samples	HQ-Derm	HQ-Inh	HQ-Ing	HI	HQ-Derm	HQ-Inh	HQ-Ing	HI
	adults				children			
SL1	2.94 × 10^–5^	2.13 × 10^–7^	4.13 × 10^–4^	4.43 × 10^–4^	1.54 × 10^–4^	3.77 × 10^–7^	3.86 × 10^–3^	4.01 × 10^–3^
SL2	4.91 × 10^–5^	3.55 × 10^–7^	6.89 × 10^–4^	7.38 × 10^–4^	2.57 × 10^–4^	6.29 × 10^–7^	6.43 × 10^–3^	6.69 × 10^–3^
SL3	5.12 × 10^–5^	3.70 × 10^–7^	7.18 × 10^–4^	7.70 × 10^–4^	2.68 × 10^–4^	6.56 × 10^–7^	6.70 × 10^–3^	6.97 × 10^–3^
SL4	5.65 × 10^–5^	4.08 × 10^–7^	7.93 × 10^–4^	8.50 × 10^–4^	2.96 × 10^–4^	7.24 × 10^–7^	7.40 × 10^–3^	7.70 × 10^–3^
SL5	2.96 × 10^–5^	2.14 × 10^–7^	4.15 × 10^–4^	4.45 × 10^–4^	1.55 × 10^–4^	3.79 × 10^–7^	3.87 × 10^–3^	4.03 × 10^–3^
SL6	2.97 × 10^–5^	2.15 × 10^–7^	4.17 × 10^–4^	4.47 × 10^–4^	1.56 × 10^–4^	3.81 × 10^–7^	3.89 × 10^–3^	4.05 × 10^–3^
SL7	1.15 × 10^–5^	8.29 × 10^–8^	1.61 × 10^–4^	1.73 × 10^–4^	6.01 × 10^–5^	1.47 × 10^–7^	1.50 × 10^–3^	1.56 × 10^–3^
SL8	4.18 × 10^–5^	3.02 × 10^–7^	5.86 × 10^–4^	6.28 × 10^–4^	2.19 × 10^–4^	5.35 × 10^–7^	5.47 × 10^–3^	5.69 × 10^–3^
SL9	5.32 × 10^–5^	3.84 × 10^–7^	7.47 × 10^–4^	8.00 × 10^–4^	2.79 × 10^–4^	6.82 × 10^–7^	6.97 × 10^–3^	7.25 × 10^–3^
SL10	1.30 × 10^–5^	9.43 × 10^–8^	1.83 × 10^–4^	1.96 × 10^–4^	6.84 × 10^–5^	1.67 × 10^–7^	1.71 × 10^–3^	1.78 × 10^–3^
SL11	1.89 × 10^–5^	1.36 × 10^–7^	2.65 × 10^–4^	2.84 × 10^–4^	9.88 × 10^–5^	2.42 × 10^–7^	2.47 × 10^–3^	2.57 × 10^–3^
SL12	1.18 × 10^–5^	8.56 × 10^–8^	1.66 × 10^–4^	1.78 × 10^–4^	6.21 × 10^–5^	1.52 × 10^–7^	1.55 × 10^–3^	1.61 × 10^–3^
SL13	3.25 × 10^–5^	2.35 × 10^–7^	4.56 × 10^–4^	4.89 × 10^–4^	1.70 × 10^–4^	4.16 × 10^–7^	4.25 × 10^–3^	4.43 × 10^–3^
SL14	1.31 × 10^–6^	9.48 × 10^–9^	1.84 × 10^–5^	1.97 × 10^–5^	6.88 × 10^–6^	1.68 × 10^–8^	1.72 × 10^–4^	1.79 × 10^–4^
SL15	1.62 × 10^–6^	1.17 × 10^–8^	2.27 × 10^–5^	2.43 × 10^–5^	8.47 × 10^–6^	2.07 × 10^–8^	2.12 × 10^–4^	2.20 × 10^–4^
SL16	9.03 × 10^–5^	6.52 × 10^–7^	1.27 × 10^–3^	1.36 × 10^–3^	4.73 × 10^–4^	1.16 × 10^–6^	1.18 × 10^–2^	1.23 × 10^–2^
SL17	1.38 × 10^–5^	9.95 × 10^–8^	1.93 × 10^–4^	2.07 × 10^–4^	7.22 × 10^–5^	1.77 × 10^–7^	1.80 × 10^–3^	1.88 × 10^–3^
SL18	4.74 × 10^–6^	3.42 × 10^–8^	6.65 × 10^–5^	7.13 × 10^–5^	2.48 × 10^–5^	6.07 × 10^–8^	6.21 × 10^–4^	6.46 × 10^–4^
SL19	5.90 × 10^–6^	4.26 × 10^–8^	8.28 × 10^–5^	8.87 × 10^–5^	3.09 × 10^–5^	7.56 × 10^–8^	7.73 × 10^–4^	8.04 × 10^–4^
SL20	1.79 × 10^–5^	1.29 × 10^–7^	2.51 × 10^–4^	2.69 × 10^–4^	9.38 × 10^–5^	2.29 × 10^–7^	2.35 × 10^–3^	2.44 × 10^–3^
SL21	3.89 × 10^–5^	2.81 × 10^–7^	5.46 × 10^–4^	5.85 × 10^–4^	2.04 × 10^–4^	4.98 × 10^–7^	5.09 × 10^–3^	5.30 × 10^–3^

### 3.5 Noncarcinogenic Risk Assessment in the Water Samples

The total mercury concentrations found in the water samples were employed to calculate the noncarcinogenic risks (HI) using the target risk quotient (HQ) index. These calculations were done by considering the potential risks of dermal absorption (ADIderw) and mercury’s oral ingestion (ADIingw), using the equations detailed in item 2.5 and the parameters shown in Table 1. The daily Hg exposure also was evaluated for adults and children. It can be seen in Table 6 that all the HI values are lower than 1.0, which indicates that the water samples analyzed do not have noncarcinogenic health risks. In addition, it can be observed that HQing > HQderm > both children and adults.^15,21,24,25^

**Table 6:** Noncarcinogenic Risk Assessment in the Water Samples from *Jacobina*, Brazil

water samples	HQ-Derm	HQ-Ing	HI	HQ-Derm	HQ-Ing	HI
	adults			children		
A1	1.19 × 10^–4^	5.02 × 10^–2^	5.03 × 10^–2^	2.72 × 10^–4^	2.34 × 10^–1^	2.35 × 10^–1^
A2	1.29 × 10^–4^	5.48 × 10^–2^	5.49 × 10^–2^	2.97 × 10^–4^	2.56 × 10^–1^	2.56 × 10^–1^
A3	1.83 × 10^–4^	7.76 × 10^–2^	7.78 × 10^–2^	4.20 × 10^–4^	3.62 × 10^–1^	3.63 × 10^–1^
A4	3.82 × 10^–4^	1.62 × 10^–1^	1.62 × 10^–1^	8.75 × 10^–4^	7.54 × 10^–1^	7.55 × 10^–1^
A5	1.21 × 10^–4^	5.11 × 10^–2^	5.13 × 10^–2^	2.77 × 10^–4^	2.39 × 10^–1^	2.39 × 10^–1^
A6	8.84 × 10^–5^	3.74 × 10^–2^	3.75 × 10^–2^	2.03 × 10^–4^	1.75 × 10^–1^	1.75 × 10^–1^
A7	7.76 × 10^–5^	3.29 × 10^–2^	3.30 × 10^–2^	1.78 × 10^–4^	1.53 × 10^–1^	1.54 × 10^–1^
A8	4.74 × 10^–5^	2.01 × 10^–2^	2.01 × 10^–2^	1.09 × 10^–4^	9.38 × 10^–2^	9.39 × 10^–2^
A9	6.69 × 10^–5^	2.83 × 10^–2^	2.84 × 10^–2^	1.53 × 10^–4^	1.32 × 10^–1^	1.32 × 10^–1^
A10	8.41 × 10^–5^	3.56 × 10^–2^	3.57 × 10^–2^	1.93 × 10^–4^	1.66 × 10^–1^	1.66 × 10^–1^
A11	3.88 × 10^–5^	1.64 × 10^–2^	1.65 × 10^–2^	8.90 × 10^–5^	7.67 × 10^–2^	7.68 × 10^–2^
A12	3.23 × 10^–5^	1.37 × 10^–2^	1.37 × 10^–2^	7.42 × 10^–5^	6.39 × 10^–2^	6.40 × 10^–2^
A13	4.10 × 10^–5^	1.74 × 10^–2^	1.74 × 10^–2^	9.39 × 10^–5^	8.10 × 10^–2^	8.11 × 10^–2^
A14	6.90 × 10^–5^	2.92 × 10^–2^	2.93 × 10^–2^	1.58 × 10^–4^	1.36 × 10^–1^	1.37 × 10^–1^
A15	4.31 × 10^–5^	1.83 × 10^–2^	1.83 × 10^–2^	9.89 × 10^–5^	8.52 × 10^–2^	8.53 × 10^–2^
A16	5.61 × 10^–5^	2.37 × 10^–2^	2.38 × 10^–2^	1.29 × 10^–4^	1.11 × 10^–1^	1.11 × 10^–1^
A17	5.61 × 10^–5^	2.37 × 10^–2^	2.38 × 10^–2^	1.29 × 10^–4^	1.11 × 10^–1^	1.11 × 10^–1^
A18	3.02 × 10^–5^	1.28 × 10^–2^	1.28 × 10^–2^	6.92 × 10^–5^	5.97 × 10^–2^	5.97 × 10^–2^
A19	2.16 × 10^–5^	9.13 × 10^–3^	9.15 × 10^–3^	4.94 × 10^–5^	4.26 × 10^–2^	4.27 × 10^–2^

### 3.6 Comparison with Previously Published Mercury Data

Santos et al. determined mercury in five sediment samples collected in *Jacobina*.^2^ The mercury concentrations for the samples with particles less than 75 μm were 0.19, 0.20, 0.41, 0.32, and 0.44 μg g^–1^. The application of the CF index results in the following CF values: 0.76, 0.80, 1.64, 1.28, and 1.76, denoting two samples with low contamination (CF < 1.0) and three samples with moderate contamination (1 < CF < 3). The evaluation with the ecological risk index at the same mercury contents establishes the Er values of 30.4, 32.0, 65.6, 51.2, and 70.4, indicating two samples with low ecological risk (Er < 40) and three with moderate ecological risk (40 < Er < 80). The same assessment performed with the mercury contents of the samples with particles smaller than 1.5 mm denotes four samples with low contamination and low ecological risk and one with moderate contamination and moderate ecological risk. This analysis reveals that the results in the present work corroborate the work published involving mercury contents in sediments from *Jacobina*.^2^

## 4 Conclusions

Gold mining activities in the city of *Jacobina* have been occurring since 1930. However, Santos et al. assessed the impact of using mercury in the extraction process of gold for the first time in 2013 when five samples of river sediments were analyzed. The results denoted a low to moderate mercury contamination.

The present work also evaluated mercury contamination in this city by collecting soil, sediment, and natural water samples. The results obtained for soil samples demonstrated a low level of contamination and low ecological risk. In addition, the human health risk index evaluated by exposure routes through dermal contact, inhalation, and ingestion showed that the soil samples did not present a noncarcinogenic risk. On the other hand, 85% of the sediment samples collected denoted low contamination and ecological risk for mercury. Analysis carried out with water samples revealed that mercury levels are below the maximum limit established by the Brazilian Government, showing low levels of contamination.

The results found in this work fully agreed with those of the work carried out by Santos.

In Brazil, there is no specific legislation to assess the risks to human health arising from chemical elements in environmental matrices. Therefore, in this work, we chose to use the noncarcinogenic indices established by USEPA, which are widely used and allow for comparing data obtained with a universal view.

Although the results indicated low mercury contamination, other research must be carried out, mainly in the city’s rivers, involving water and sediment samples and animal and plant matrices that tend to bioaccumulate mercury from bodies of water.
